# Exploring Physician Perspectives on Using Real-world Care Data for the Development of Artificial Intelligence–Based Technologies in Health Care: Qualitative Study

**DOI:** 10.2196/35367

**Published:** 2022-05-18

**Authors:** Martina Kamradt, Regina Poß-Doering, Joachim Szecsenyi

**Affiliations:** 1 Department of General Practice and Health Services Research University Hospital Heidelberg Heidelberg Germany

**Keywords:** artificial intelligence–based solutions, data donation, qualitative research, Germany, artificial intelligence, requirement analysis, physician perspective, real-world data, big data, data pool, interview, qualitative

## Abstract

**Background:**

Development of artificial intelligence (AI)–based technologies in health care is proceeding rapidly. The sharing and release of real-world data are key practical issues surrounding the implementation of AI solutions into existing clinical practice. However, data derived from daily patient care are necessary for initial training, and continued data supply is needed for the ongoing training, validation, and improvement of AI-based solutions. Data may need to be shared across multiple institutions and settings for the widespread implementation and high-quality use of these solutions. To date, solutions have not been widely implemented in Germany to meet the challenge of providing a sufficient data volume for the development of AI-based technologies for research and third-party entities. The Protected Artificial Intelligence Innovation Environment for Patient-Oriented Digital Health Solutions (pAItient) project aims to meet this challenge by creating a large data pool that feeds on the donation of data derived from daily patient care. Prior to building this data pool, physician perspectives regarding data donation for AI-based solutions should be studied.

**Objective:**

This study explores physician perspectives on providing and using real-world care data for the development of AI-based solutions in health care in Germany.

**Methods:**

As a part of the requirements analysis preceding the pAItient project, this qualitative study explored physician perspectives and expectations regarding the use of data derived from daily patient care in AI-based solutions. Semistructured, guide-based, and problem-centered interviews were audiorecorded, deidentified, transcribed verbatim, and analyzed inductively in a thematically structured approach.

**Results:**

Interviews (N=8) with a mean duration of 24 (SD 7.8) minutes were conducted with 6 general practitioners and 2 hospital-based physicians. The mean participant age was 54 (SD 14.1; range 30-74) years, with an average experience as a physician of 25 (SD 13.9; range 1-45) years. Self-rated affinity toward modern information technology varied from very high to low (5-point Likert scale: mean 3.75, SD 1.1). All participants reported they would support the development of AI-based solutions in research contexts by donating deidentified data derived from daily patient care if subsequent data use was made transparent to them and their patients and the benefits for patient care were clear. Contributing to care optimization and efficiency were cited as motivation for potential data donation. Concerns regarding workflow integration (time and effort), appropriate deidentification, and the involvement of third-party entities with economic interests were discussed. The donation of data in reference to psychosomatic treatment needs was viewed critically.

**Conclusions:**

The interviewed physicians reported they would agree to use real-world care data to support the development of AI-based solutions with a clear benefit for daily patient care. Joint ventures with third-party entities were viewed critically and should focus on care optimization and patient benefits rather than financial interests.

## Introduction

In recent years, research on artificial intelligence (AI)–based technologies in medicine has been proceeding rapidly [1,2]. It is assumed that AI will change medicine, health care, and the future role of physicians [3]. Among the key practical issues to be solved are the sharing and release of real-world care data prior to the development of AI-based solutions and implementation into existing clinical practice. A precondition for the development, widespread use, and acceptance of AI-based technologies in medicine and health care is an adequate, structured, and controlled data pool derived from daily patient care, with a continuous data supply to provide for the ongoing training, validation, and improvement of AI-based solutions [4]. Meeting this precondition would greatly benefit a widespread implementation of AI-based technologies and achieve improvements for health care delivery, the quality of patient care, and diagnosis and treatment outcomes. An automated and consolidated flow of deidentified real-world care data, ideally from multiple facilities in clinical and ambulatory settings, into a data pool is perceived to be a possible solution to overcome the challenge of providing a sufficient data volume for secondary use in research and implementation [4].

An example of a freely accessible data pool for AI-based research is the MIMIC-III (Medical Information Mart for Intensive Care) database in the United States [5]. Additionally, a common European Health Data Space is envisioned to facilitate the exchange of and access to different types of health data, including electronic health records, genomics data, and data from patient registries, to support health care delivery, along with the secondary use of data for health research and policy-making [6]. Several Nordic countries in Europe, such as Sweden, already offer disease-specific registries providing real-world patient data that enables researchers to analyze complex phenomena [7]. In Germany, efforts to meet the challenge of providing an adequate data pool for research and the development of AI-based technologies still need to gain momentum, though the availability of data pools derived from real-world patient care is expected to increase in the near future via the now legally regulated use of electronic patient records [8,9] and a government initiative to support the development and establishment of a nationwide research health data center [10]. The German Medical Informatics Initiative (MII) was established to make data from health care and research more useful and meaningful and develop conditions to make routinely collected clinical health care data available for medical research purposes [11,12]. The aim of the initiative is to optimize research and patient care options through innovative information technology solutions that facilitate the cross-institutional exchange and use of health care, clinical, and biomedical research data. In several large consortia, German university hospitals at more than 30 sites work together with research institutions, health insurers, private entities, and patient representatives to establish the conditions for this exchange. Both legal and ethical conditions need to be considered when governing who will use the data and for which purposes [13]. However, to date, real-world care data are not centrally available and accessible for research purposes, including technical developments. Instead, in most cases physicians, health care facilities, or patients need to give consent for data use, even if the data were deidentified [13]. There is no uniform legal status in Germany on how and for which secondary research purposes health care data can be used [14,15]. In some German states, it is possible to use clinical health care data under certain requirements for secondary purposes, like research, without explicit patient consent. However, the data still remain within each institution and are not centrally stored with uniform quality and privacy standards. Moreover, the distinct separation of different health care settings and restrictive data privacy regulations in Germany compared to other European countries pose a challenge for the exchange and linkage of health care data [16]. For third-party entities that develop AI-based technologies for health care, access to sufficient real-world care data for training and validation remains scarce.

The Protected Artificial Intelligence Innovation Environment for Patient-Oriented Digital Health Solutions (pAItient) project aims to meet this challenge by creating a large data pool that feeds on data donated by primary care and hospital physicians, patients, and health care institutions. Prior to building this data pool, physician perspectives regarding data donation for AI-based solutions need to be studied. Insight into the attitudes and concerns of physicians working in different settings regarding data donation is central for the development and implementation of this concept into real-world health care. Therefore, the aim of this study was to explore physicians’ perspectives on providing and using real-world care data for the development of AI solutions in health care in Germany.

## Methods

### Study Design

This study is a part of the pAItient project, which aims to develop and test a solution for providing real-world care data for AI development, validation, and implementation. In the first phase of problem identification, a requirements analysis was conducted to specifically gain insight into physicians’ perspectives on providing and using real-world care data for the development of AI solutions in health care in Germany. In the exploratory approach, semistructured, guide-based, and problem-centered interviews [17] were conducted via telephone with physicians working in clinical or ambulatory settings. The study-specific interview guide was developed by the interprofessional research team (Health Services Research, Public Health, and Information Technology) at University Hospital Heidelberg and was based on a literature research and predefined, project-specific research questions. Each draft of the interview guide was discussed within the interprofessional research team to reach consensus about the content. The interview guide focused on the exploration of knowledge and attitudes regarding AI in general and patient care, as well as the secondary use of health care data in AI applications (see Multimedia Appendix 1 for a translated English version). A sociodemographic questionnaire was developed and used to collect participant characteristics. Field notes were taken after each interview and discussed within the research team. All interviews were audiorecorded and transcribed verbatim by experienced support staff at the Department of General Practice and Health Services Research, University Hospital Heidelberg. All transcripts were proofread by the research team and amended where applicable. The transcripts were not returned to the participants.

### Ethics Approval

This study received ethical approval by the medical ethics committee of the Medical Faculty of Heidelberg University (S-241/2021).

### Recruitment

During the first recruitment phase, a convenience sample of 33 general practitioners (GPs) and 16 hospital-based physicians was approached using known contacts in academic teaching practices and personal contacts in hospital departments in Baden-Wuerttemberg. Given the explorative nature of this study and based on prior experiences and empirical guidance [18], a targeted sample size of 10 was deemed appropriate and sufficient to identify broad categories and themes of interest in the collected data and subsequently defined in the (unpublished) study protocol. All potential participants received an invitation to participate and written information about the aim of the study via post or email. In the second recruitment phase, a reminder was sent to all physicians who had not replied after 3 weeks (n=28). Since the COVID-19 pandemic was still ongoing and potential recruits signaled that they could only participate after the pandemic was over, no further recruiting waves were initiated. Considerations of gender, work experience, or specialty balance could not be applied due to the nature of convenience sampling. Physicians who were interested in participation returned a contact form and indicated a preferred date and time for the interview. All participants gave written consent prior to the interview. All interested physicians were included in the study. No reimbursement was offered.

### Data Analysis

Qualitative data were analyzed in a structured content analysis [19] and categorized into relevant themes emerging from the data. Subsequently, 2 experienced researchers (MK and RPD) inductively identified the main categories and subcategories from the data, discussed and approved the coding, and assessed thematic saturation in close consultation with the study team. Organization and management of all text data was performed using MAXQDA Plus 2018 software (release 18.2.4; VERBI GmbH). Participant characteristics were analyzed using SPSS software (version 27; IBM Corp).

## Results

### General Characteristics of the Interviews

Interviews (N=8) with a mean duration of 24 (SD 7.8) minutes were conducted with 6 GPs and 2 hospital-based physicians in May and June 2021 by 2 experienced female interviewers (MK and RPD) who were members of the study team. All phone interviews were conducted either at the interviewer’s workplace or home office. No other persons were present during the interviews. There were no prior connections between the participants and researchers. All participants could ask questions about the study before the interview and verbally confirmed their consent for participation. The inductive analysis identified a wide range of themes in broad categories, thus providing an indication of data adequacy. The findings outlined below reflect the physicians’ perspectives on donating and using real-world care data for the development of AI solutions in health care and their general expectations and concerns regarding AI use in medicine. Represented are findings referring to general individual views, attitude toward including commercial partners in development phases, perceived prerequisites for consent to data donation and use, and perceived potential benefits of AI-based health care innovations. Textbox 1 gives an overview of the main categories and subcategories identified from the collected data material. Extracted quotes supporting key statements are included for illustration, and the participant number and transcript position are provided. All quotes were translated into English with due diligence.

**The main categories and subcategories identified from the collected data material.**

**Data use for artificial intelligence (AI) solutions development**

- Attitude in general and toward including partners
- Consent and conditions for data donation and use

**AI in medicine**

- Potential benefits and expectations
- Concerns

### Participant Characteristics

Of the participants, 88% (7/8) were male. All participants could self-rate affinity toward modern information technology (5-point Likert scale from 1=very low to 5=very high affinity). Table 1 describes the participant characteristics.

**Table 1:** Participant characteristics (N=8).

Characteristic		Value
**Care setting, n (%)**		
	General practice	6 (75)
	Hospital	2 (25)
**Medical specialty, n (%)**		
	General practitioner	6 (75)
	Surgeon	2 (25)
**Gender, n (%)**		
	Female	1 (12)
	Male	7 (88)
Age (years), mean (SD); range		54 (14.1); 30-74
Experience as a physician (years), mean (SD); range		25 (13.9); 1-45
Affinity toward modern technologies^a^, mean (SD); range		3.75 (1.1); 1-5

^a^From 1=very low to 5=very high affinity.

### Data Use for AI Solutions Development

#### Attitude in General and Toward Including Partners

Participating physicians were open-minded about the idea of donating real-world care data for AI solutions development. They considered using real-world care data necessary to gain new knowledge and support evidence-based medicine, physicians’ decision-making, and the development of new technologies to be used in clinical practice. Participation in the development of new AI-based technologies was seen as a chance to develop and use a target-oriented, user-centered product for patient care in a reasonable amount of time. Physicians also viewed this as a possibility for low-threshold research contributions.

This is indispensable, so if you want to develop artificial intelligence in the medical context, you cannot do it without patient data.

Physician 02, #32

If this could be implemented into the regular administrative software and one could enable a direct data flow with relatively low expenditure, and also in such a transparent way so no misuse was possible, then I think collecting gigantic data volumes in GP practices would be low threshold. And I believe, lots and lots and lots would be participating.

Physician 07, #39

Physicians mentioned that they would agree to donate data derived from their daily patient care if prerequisites were met. It was emphasized that even in research contexts, transparency about actual data use and strict data deidentification were of priority. High security standards and supervision of data use were expected to prevent misuse. This was mentioned especially in the context of the involvement of third-party entities with economic interests. Tight control of data use, possibly government-regulated, was suggested, and transparency about involved parties and their interests and goals was considered necessary to mitigate potential conflicts of interest (profit-oriented vs common welfare). Voluntary patient and physician participation was seen as an important prerequisite as well as requiring minimal additional efforts by physicians, similar to an automatically generated data flow form for their practice’s administration system.

...it must be ensured that data is deidentified...Overall, the process must be transparent, you must always be able to understand what is done with the data and how it is processed. Exactly...who processes the data, what research projects are being carried out...

Physician 03, #40

...good informing would have to happen...who is involved, who does the developing, who are the potential funders of such development. This would be very important to have relative transparency about, I believe. Because this is something that is relatively important...

Physician 08, #30

#### Consent and Conditions for Data Donation and Use

Sufficient information and transparency about data use and security, deidentification, and intended purpose were pointed out as prerequisites for consenting to data donation for AI solution development and use in the provision of health services. Physicians’ consent was seen as mandatory for data donation, and patient consent was discussed to be necessary only in specific cases, such as rare diseases. In general, donating data from real-world care processes was classified as being voluntary on the physician side, bound to specific purposes and suitable research questions. Again, adequate data protection and deidentification were mentioned as being mandatory. Purposeful and proven benefit in terms of patient and common welfare and a nonprofit use were further perceived as prerequisites for consent. The economic interests of third-party entities involved in development were expected to be of secondary importance in related research projects.

...I would have a strange feeling if I knew data from various practices were being tapped and I had no idea what was happening with them. So, I think that would be fundamentally the wrong way to go about it.

Physician 04, #28

There has to be an adequate research question defined for the research project and implementation of the study, data analysis and also further data use must be transparent.

Physician 02, #47

### AI in Medicine

#### Potential Benefits and Expectations

Regarding patient care, the physicians saw several potential benefits of AI use when based on real-world care data. They mentioned aspects regarding the efficiency of health care provision—that more time could be left for direct patient interaction and they expected their decisions to be supported by AI. They also reflected on a potential increase of evidence-based knowledge by using real-world care data derived from their own medical specialty and assumed there would be fewer restrictions on transferability to their own practice than using data from other medical fields.

Exactly, more time for patients, then better decisions to improve mortality and morbidity of patients. Exactly, I believe these are the two most important aspects.

Physician 03, #12

Simply because a GP’s work is so incredibly complex, and I believe artificial intelligence could also support our work by optimizing our time.

Physician 07, #12

Physicians expected strong support for daily patient care when AI technology is in place. They focused on the potential of a decreased workload, particularly in complex areas such as medication, yet final decisions were considered to remain with the physician. Another assumed benefit was that “a knowledge leap” (Physician 01, #10) would occur when enormous data volumes are analyzed.

I believe these can be supporting systems...which analyze CT data in a structured way, but certainly do not substitute the experienced radiologist who assesses them.

Physician 02, #18

#### Concerns

Regarding the involvement of third-party entities with economic interests, all physicians contemplated the potential conflicts of interest. Their concerns were related to endeavors that potentially have a strong commercial interest instead of a focus on patient and common welfare and care optimization. The fear of potentially being replaced by technology was discussed by one participant. Physicians expressed that from their perspective, professional experience and diligence cannot be substituted by AI-based technology, particularly regarding their knowledge about individual patients and other possible medical findings. Further concerns referred to potential mistrust in data derived from sources unknown to the physicians and technical feasibility limits.

There has to be good information...Who contributed to the development and possibly is a funder of this development. That would be very important to be relatively transparent, I suppose.

Physician 08, #30

## Discussion

### Principal Findings

All participants in this study reported they would support the development of AI-based solutions in research contexts by donating deidentified data derived from daily patient care if subsequent data use was made transparent to them and their patients and the benefits for patient care were clear. Contributing to care optimization and efficiency were cited as motivation for potential data donation. Concerns regarding workflow integration (time and effort), appropriate deidentification, and the involvement of third-party entities with economic interests were discussed. The donation of data in reference to psychosomatic treatment needs was viewed critically.

### Comparison With Prior Work

Using real-world care data and AI-based assistance systems can support prevention, early diagnostics, and individualized treatment in the future to facilitate improved outcomes and the discovery of new medical correlations and innovative preventive approaches that optimize health care [20,21]. AI-based systems can also facilitate more differentiated treatment methods and improved aftercare, thus assisting physicians and the nonphysician health care workforce by providing optimized patient care while easing their workload [20]. The development of these systems for research and health care requires a large volume of data, and data security is required to build and maintain trust in them for health care providers and patients alike. Therefore, exploring their respective perspectives on AI-based solutions in health care is just as essential as creating and implementing necessary regulation that defines the conditions and boundaries of secondary use of real-world health care data in general. Such structures have already been created in several other countries [22]. In Scandinavian countries, social and health care data of each citizen are connected with a unique identification number. For example, Finland has established legal regulations for the secondary use of social and health care data from different sources with the Finnish Social and Health Data Permit Authority [23]. In contrast, legal regulations for the secondary use of health care data in Germany are fragmented, and strict data privacy regulations, which still follow the principles of data anonymization and minimization, impede the secondary use and linkage of health care data from different sources [15,16]. Obtaining informed consent for the use of health care data by the data owner is still common in Germany to meet the legal requirements for research. The MII in Germany was created to bridge this gap, and it aims to establish a nationwide infrastructure that enables the donation and sharing of digitally available health care data for biomedical research [12]. Prior research identified the challenges to a widespread secondary use of real-world care data in Germany, which include a traditionally high weighting of data protection and concerns about sharing and innovatively using such data [24]. These challenges are mirrored in our findings, particularly the strong emphasis the participants in our study put on transparency about the use, security, and deidentification of donated data, and the intended research purpose. The implementation of the broad consent model, as developed and approved by one of the MII expert working groups, might mitigate some of these concerns since the model will facilitate broad and pre-use transparency, allowing for consent withdrawal based on project-specific information provided in digital formats prior to actual project implementation [25]. In addition to the MII, which focuses on data sharing and the provision of data for research purposes in the clinical setting, there are further initiatives in Germany aimed at the sharing and provision of data in the primary care setting [16].

Besides transparency concerns, the physicians in our study also contemplated potential changes the use of AI-based technology might bring for their profession. These concerns were also considered in a Science for Policy report compiled by the Joint Research Centre for the European Commission. The report predicts that the incorporation of AI-based technologies into medical practice will trigger substantial changes in health care and medicine across medical, scientific, and technical grounds, as well as in workflows, clinical pathways, management, and the physician-patient relationship [26]. The report also covers ethical and social issues related to using AI-based systems in health care and medicine that were contemplated by the physicians in our study as well. It states that these issues coincide with some of the urgent priorities for the coming decade as defined by the World Health Organization in 2020, including “earning public trust” and “protecting people from dangerous products” [27]. The report also stipulates that to evaluate a patient’s clinical situation and treatment options, the integrated analysis of a qualified, trained, and real physician is necessary, a perspective clearly shared by the physicians in our study who viewed AI-based solutions as supporting tools rather than a decision-making authority.

The benefits that AI-based technologies can offer for health care are indivisibly linked to the sharing of health care data among different entities; however, resources for analyzing large data sets and developing new (AI-based) technologies for health care delivery by public research institutions or the government might be limited. The involvement of third-party entities with potential commercial interest can be a key to realizing the potential linked with large data sets and AI efforts. However, studies have shown that there are concerns about the use of health care data by third-party entities and that data sharing can lead to a deterioration of patient trust [28-30]. For instance, a recent survey regarding secondary use of health data among the German population assessed a generally positive attitude toward the use of personal health data for research purposes, and a mostly disapproving attitude regarding data use for commercial purposes, such as in the pharmaceutical industry [13]. In further research, patients in the Netherlands as well as German citizens were surveyed regarding the secondary use of health data, different options for consenting to data use, and data donation [31]. The findings indicated a willingness to permit secondary data use depending on the beneficiary institution or purpose, as well as a wide acceptance of broad consent in Dutch patients. The majority of the 1006 (78.8%) surveyed German citizens approved of anonymized data donation from their digital health records and the sharing of these data with third-party entities for medical research. Only a small minority of participants disagreed, mainly because of worries about data security [31]. A survey assessed that the US public is more comfortable sharing health data with third-party entities for patient purposes than business purposes [32]. An increase of public comfort in sharing health care data with third-party entities might be acheived by emphasizing patient-centered benefits and transparent communication about protective actions regarding data privacy and deidentification [29,32,33]. The physicians in our study strongly supported these views and repeatedly emphasized that transparency about the purpose of data use was mandatory to facilitate and increase comfort in sharing data, especially when third-party entities were to be involved. In general, they were supportive of donating real-world health care data through an automated and consolidated flow into a deidentified data pool for secondary use in research and implementation [4]. They were also interested in the potential benefits that analyzing large sets of routinely collected health care data might offer to them and their patients. Nevertheless, the physicians clearly highlighted their perspective regarding the purpose of data use, saying that such endeavors should primarily benefit patient outcome, health care delivery, or society. Statutory and best practice guidelines will need to accommodate these considerations so that physicians and patients can donate real-world health care data while empowered with knowledge and according to their beliefs.

In summary, the findings of this interview study support the need for better access to real-world health care data with uniform rules and legal regulations. Physicians from different settings interviewed in this study seem to be open-minded toward the concept of using health care data for research purposes and the development of new AI-supported technical tools. The concept of a new, large data pool should consider the inclusion of health care data from different institutions and settings and that the way data is transferred into the data pool should not add to physicians’ workloads. Moreover, the use of data needs to follow the principle of transparency, especially if third-party entities are involved. In accordance with findings from other studies, a concept for the donation, storage, and use of health care data in Germany should also focus on increasing public comfort in sharing health care data with third-party entities.

### Strengths and Limitations

This qualitative study was guided by methodological strategies aimed at minimizing potential bias and reducing the risk of losing relevant content. Conducting the interviews via telephone ensured minimal added burden to the participants. During the analysis, the inductive approach facilitated the identification of relevant themes and a high intercoder congruence was achieved, reflecting a reliable classification of the data. The density of the generated data allowed for a thorough analysis and sufficient illustration of the inductive categories, pointing to thematic saturation and effective convenience sample size as indicated by empirical guidance [18]. The reporting of this study followed the Consolidated Criteria for Reporting Qualitative Research (COREQ) guideline [34].

However, a limitation of potential selection bias has to be considered, since a pre-existing motivation for the dissemination of personal attitude, opinion, and experience might have been present. It is also possible that socially desirable answers were given. Further, female physicians, who were underrepresented in this sample, might have shared different or additional perspectives. To limit bias, the interviewers continuously established rapport with all participants and repeatedly provided reflection-enabling prompts. The discussion of perceived tendencies and the refinement of adequate approaches was facilitated by debriefings in regular research team meetings during data collection and analysis.

### Conclusions

The physicians interviewed in this study reported they would donate real-world care data for secondary use in research contexts and the development of AI-based solutions with clear benefits for care optimization. The remaining identified concerns would need to be adequately addressed through national and international regulations for data sharing and options for consenting to provide a solid foundation for the development of new assistive AI-based solutions.

## Appendix

Multimedia Appendix 1 Translated thematic interview guide for physicians in English.

## Abbreviations

AI: artificial intelligence
COREQ: Consolidated Criteria for Reporting Qualitative Research
GP: general practitioner
MII: Medical Informatics Initiative
MIMIC-III: Medical Information Mart for Intensive Care
pAItient: Protected Artificial Intelligence Innovation Environment for Patient-Oriented Digital Health Solutions
