# Supplementary material for: Exploring Physician Perspectives on Using Real-world Care Data for the Development of Artificial Intelligence–Based Technologies in Health Care: Qualitative Study
Source: JMIR Form Res. 2022 May 18;6(5):e35367. doi: 10.2196/35367 (PMC9161099; doi:10.2196/35367)
Supplement: Multimedia Appendix 1 [file formative_v6i5e35367_app1.pdf]

Kamradt M, Poß-Doering R, Szecsenyi J (2022) Exploring physician perspectives on using real-world care data for the development of artificial intelligence-based technologies in healthcare: qualitative study. JMIR Form Res 2022;0(0):e0. doi: 10.2196/35367

Appendix 1: Translated thematic interview guide for physicians

Knowledge and attitudes

(1) General associations with the term AI
(2) Known solutions that use AI in everyday life
(3) Known AI-solutions that support daily patient care
(4) Expectations if data from daily patient care is used for development of AI-based solutions
(5) Further fields for application
- benefits
- disadvantages
- concerns
- How to be met?
- Which decisions would be left to AI?
- Where not to rely on AI?

Secondary usage of data in AI applications

(6) Perspectives on using real world care data for development of AI-based technology in healthcare
(7) Perspectives on including commercial partners
(8) Which data could be used for development of AI solutions?
- Which data would not be suitable?

(9) How to inform about usage of provided data
- consent?

(10) Pre-conditions for positive perspective on secondary data usage for AI solutions

Reflection

Perspectives to add? What was missing?

Final

Thank you very much for your participation and for sharing your thoughts and perspectives!
